# Emotional Intelligence Components in Alcohol Dependent and Mentally Healthy Individuals

**DOI:** 10.1155/2015/841039

**Published:** 2015-03-29

**Authors:** Arash Mohagheghi, Shahrokh Amiri, Seyedreza Mousavi Rizi, Salman Safikhanlou

**Affiliations:** Research Center of Psychiatry and Behavioral Sciences (RCPBS), Tabriz University of Medical Sciences, Tabriz 51677, Iran

## Abstract

*Objective*. Emotional intelligence might play an important role in the onset and persistence of different psychopathologies. This study investigated the relationship between emotional intelligence and alcohol dependence. *Methods*. In this case-control study, participants included alcohol dependent individuals and mentally healthy inpatients. Each group consisted of 40 individuals (male/female: 1). The diagnosis was based on the criteria of the DSM-IV-TR using the Structured Clinical Interview for DSM-IV (SCID-IV). All the participants completed Bar-On emotional intelligence test. *Results*. 20 males and 20 females were included in each group. Mean age of alcohol dependent participants and controls was 31.28 ± 7.82 and 34.93 ± 9.83 years in that order. The analyses showed that the alcohol dependent individuals had a significant difference compared with the control group and received lower scores in empathy, responsibility, impulse control, self-esteem, optimism, emotional consciousness, stress tolerance, autonomy, problem-solving, and total score of emotional intelligence components. *Conclusion*. Patients with alcohol dependence have deficits in components of emotional intelligence. Identifying and targeted training of the individuals with lower scores in components of emotional intelligence may be effective in prevention of alcohol dependence.

## 1. Introduction

Substance use and dependence have been serious health problems for years. Risks of life-time alcohol-related disorders in males and females are 15% and 8–10%, respectively. Genetic and environmental factors are responsible for 60% and 40% of the risk. Patients with substance dependence have a higher chance of becoming dependent on a second substance. An extensive monitoring called NSDUH (National Survey on Drug Use and Health) was carried out in USA on all the substance related disorders reporting alcohol as the most commonly used substance after tobacco [1]. Stress is related to alcohol consumption while further stress is generated after alcohol consumption. Stresses are also accompanied by excessive alcohol consumption [1]. There has been a challenge of finding preventive and effective treatment methods for alcohol dependence.

In 1990, Kun and Demetrovics defined emotional intelligence (EI) for the first time as “the ability to understand and examine feelings and emotions of oneself and others; therefore, an individual takes advantage of the data further and exhibits better practices and behaviors” [2]. Studies showed that there is a relationship between emotional intelligence and psychosomatic health. “Emotions” have been the dominant components in theories and treatments of mental disorders since late 19th century. Freud considered the role of emotions in substance dependence and believed that there is a way out of fear, pain, and despair in the psychological background of drug abuse. Studies showed a lower level of EI in the individuals who consume alcohol and smoke; however, consumption of other illegal drugs is also common in those who have a low EI [2].

The study of EI started about 20 years ago; however, a correct realization of it and its effect on individuals during their lives has not been come to yet [3]. EI determines how emotions direct an individual toward life objectives. The relationship between EI and life skills shows that a higher level is accompanied with a better problem-solving skill and adaptation as well as a lower level of anxiety [4].

Numerous studies have evaluated different aspects of EI like gender difference and indicate that the difference is not considerable; however, few studies have been conducted on the consumption of drugs and alcohol among females and males [5]. EI is effective in alcohol use and negative affection tendency. Desire for alcohol as a withdrawal symptom at the beginning of treatment is higher in the alcohol dependent patients with low level of EI [6].

Despite religious believes, alcohol consumption has been increased in Iran during recent years, but few studies have been conducted on the effect of emotional intelligence on alcohol dependency. This study aimed to investigate the relationship between EI and alcohol dependence and hypothesized that patients with alcohol dependence would have a lower level of EI compared to mentally healthy individuals. We also evaluated components of EI and their possible relation to gender and age of alcohol consumption in alcohol dependent patients.

## 2. Materials and Methods

### 2.1. Participants

This was a case-control study. Alcohol dependent individuals were continuously enrolled from the outpatient referrals to the Psychiatric Clinics of Razi University Hospital, Tabriz, Northwest of Iran. Admitted patients hospitalized in the same hospital fulfilling the criteria were also enrolled continuously.

The control group members were selected from the inpatients hospitalized in the internal and neurological wards of Razi Hospital as well as referrals to the clinics. They were matched in terms of age, gender, level of education, and occupation.

Psychiatric condition of all of the patients was evaluated by a psychiatric interview by a psychiatrist based on the DSM-IV criteria. All of the participants were enrolled after giving written informed consent. The inclusion criteria for the alcohol dependent group were the diagnosis of alcohol dependence age above 18 years old and minimum level of education of diploma. The exclusion criteria were any major psychiatric disorders for the alcohol-consuming group (except for alcohol dependence) and control group, organic brain disorders, and intellectual disability (based on the patients' history, physical examination, and medical records).

### 2.2. Measures

#### 2.2.1. Bar-On Emotional Intelligence Questionnaire

Bar-On scale was used for studying EI. Bar-On Emotional Intelligence Questionnaire was started in 1980 by posing the question, “*Why are some people more successful than others in life*?” The questionnaire includes 117 questions and 15 scales, which was executed and normalized on 3831 individuals using Bar-On. Test answers were arranged on the 5-point Likert scale (*completely agree, agree, somehow agree, disagree, and completely disagree*). The test scales include emotional self-awareness, self-expression, self-esteem, self-actualization, autonomy, empathy, social responsiveness, interpersonal relations, realism, flexibility, problem-solving, stress tolerance, impulse control, optimism, and happiness. Persian version of the questionnaire was executed in Iran on 500 males and females at different ages (18–40) and Samoei (2003) confirmed its validity and reliability in Iran [7].

#### 2.2.2. Structured Clinical Interview for DSM-IV (SCID-IV)

This is a structured clinical interview based on DSM-IV criteria, which is used for diagnosing axis I and axis II psychiatric disorders. Its execution needs clinical judgment of an interviewer on the replies of an interviewee; therefore, the interviewer should have clinical knowledge and experience on psychopathology. In addition, one of the objectives of creators of this tool is to design an interview, which is structured and simple for clinical specialists to use [8]. SCID-IV has been used more than any other forms of psychiatric diagnostic interviews in psychological studies and has a global credibility. The reliability and validity of Persian version of SCID-IV have been assessed in Iran, showing an acceptable reliability and validity [8]. Here, SCID-IV was used for the diagnosing of alcohol dependence and exclusion of other psychiatric disorders of an attendant.

### 2.3. Statistical Methods

All statistical analyses were performed using SPSS version 19. The data are reported by means of descriptive statistics (i.e., mean, standard deviation, frequency, and percentage) and inferential statistics methods including Student's *t*-test and Pearson correlation coefficient. Differences were considered significant by a *P* value of less than 0.05.

## 3. Results

Gender distribution was equal in the two groups and 20 males and 20 females were included in each group.

Table 1 shows the demographic characteristics of participants in the two groups. Mean age of the alcohol dependent individuals was 34.93 ± 9.83 years and the mean age of controls was 31.28 ± 7.82 years (*P* = 0.07). Components of EI in alcohol dependent group and controls are showed and compared in Table 2.

The results show that the alcohol dependent group and mentally healthy individuals (control group) have a significant difference in problem-solving, autonomy, stress tolerance, emotional self-awareness, optimism, self-esteem, social responsiveness, empathy, impulse control scales, and total score of the emotional intelligence test. As described in Table 2, controls scored higher in these scales compared to the alcohol dependent group. The greater effect size was related to social responsiveness, followed by empathy and then the total score of EI.

Although scores of happiness, self-actualization, realism, interpersonal relations, flexibility, and self-expression scales were also higher in the control group, this difference did not reach the significance.

We also examined any correlation between components of EI and the age of alcohol consumption. As described in Table 3 the only significant relationship between EI and the age to start alcohol consumption is related to the stress tolerance scale of the alcohol dependent individuals.

Table 4 shows a significant difference between males and females with alcohol dependence group in regard to emotional self-awareness, optimism, and flexibility scales of the EI with the superiority in favor of males.

## 4. Discussion

Emotional intelligence can be considered as the final progress in the field of emotion and recognition, which is very important in describing and interpreting emotions and feelings. It includes a series of emotions, social knowledge, and the abilities that direct and strengthen our overall capacity to respond to environmental factors and pressures and improve performance in four fields of self-awareness, social awareness, relationship management, and self-management. The current study compared components of EI in patients with alcohol dependence to mentally healthy individuals. As EI is a pervasive state in each person, it may play an etiological role as predisposing or perpetuating psychopathologies like substance dependence and such patients may benefit from targeted interventions to prevent substance related disorders.

The study showed that the scores of majority of EI components have a significant difference between alcohol dependent and mentally healthy individuals, in favor of the normal individuals with one of the scales being the total score of EI. The studies in Canada, Poland, and America indicate that about half of drug and alcohol dependent individuals have low EI [9]. However, in an inconsistent study compared to ours, Saklofske et al. (2007) proved that there is no relationship between alcohol consumption and EI [10].

According to the results, emotional intelligence of alcohol dependent individuals scored significantly lower than the control group. Similar studies in this concern include reports by Goleman (2004) and Janati et al. (2010) [11, 12]. Goleman (2004) concluded that low EI is related to issues such as violence, depression, crime, and addiction, as they are all caused by an individual's inability to cope with emotions [11]. Janati et al. (2010) proved a significant relationship between EI and likelihood of drugs addiction among students [12].

Earlier studies reported a negative relationship between EI and drug addiction [13–15] such as alcohol, tobacco, and cigarette [16–18]. Our study examined the relationship between emotional intelligence scales and the age to start alcohol consumption and only stress tolerance scale had a negative relationship.

This research showed that alcohol dependent patients have a low EI. This result is consistent with reports of Trinidad and Johnson (1995), Trinidad et al. (2008), Goleman (2004), Parker (2002), Taylor and Kinderman (2001), and Zardkhaneh et al. (2008) [11, 16, 18–21]. Moreover, we evaluated components of EI in detail. This study found a significant difference between members of control group and members of alcohol dependent group regarding the empathy subscale of EI. This finding is consistent with the earlier studies and indicates that low empathy may impel people toward alcohol consumption and other drugs and might have a role in amount of consumption as described by earlier studies. It is reported that individuals with low EI are less likely to empathize with others [22] and consume more alcohol [23].

Self-esteem is another component of the EI questionnaire. Our study showed a significant difference between controls and alcohol dependent groups. This result is compatible with Bermas (2004). Bermas pointed out that addictive patients have an overall lower score at self-esteem scales [24]. Alcohol consumption is used as a self-medication in some cases to improve confidence. As self-esteem is mostly built during early adulthood, targeting such problem may prevent starting alcohol consumption as a self-medication. From another point of view, abuse of alcohol as a solution to problems could be interpreted as a deficit in problem-solving skill. Previous studies show that addicts have problems with their self-care such as inability to tolerate stress and daily efforts at problem-solving [25]. Here, normal individuals were more successful than the alcohol dependent individuals in problem-solving which is consistent with the mentioned studies.

Evidence indicates that those who become dependent on drugs and/or alcohol are not able to understand and speak about their feelings [9]. They are also unable to use their feelings as sensory symptoms or to cope with their old feelings and as a result they look for drugs. It is proposed that they use drugs to remove their ambiguous and unknown stresses and discomforts and then relax. Therefore, they only have an ambiguous understanding of their feelings and they usually attribute them to irrational factors in their body or environment [9]. Our study also discovered similar findings. The alcohol dependent individuals received lower scores at self-awareness scale. It means that they have a weak emotional awareness. This deficit was more obvious among females.

On the other hand, interpersonal subscale was not significantly different between the two groups of participants though alcohol dependent individuals received lower scores. Based on the above-mentioned items and the earlier studies, the individuals with drug abuse have a higher rate of isolation; that is, they encounter problems in their interpersonal relations and obtain lower scores [26]. Goleman et al. quoted from Parsa, Benjamin and Wulfert (2003), and Clark et al. (2005) that establishing interpersonal relations may considerably improve capacities of an individual as a kind of social support against environment pressures [27, 28].

Higher emotional intelligence is accompanied with superior mental ability for processing social information [29]. Such ability may help individuals to have a better understanding of negative and harmful consequences and act more successfully against mental and social pressures for drug consumption. This is reflected in the present results as the individuals with alcohol dependence have a lower level of stress tolerance. The age of starting alcohol consumption was also significantly lower in these patients.

Another component of EI which is impulsivity was higher in female patients but the difference did not reach the significance. Therefore, our findings are consistent with other studies. The study of Benjamin and Wulfert (2003) in this concern specified that women had a high level of impulsivity in one of the addictive behaviors (such as alcohol dependence) [27]. Clark et al. (2005) also concluded that impulsivity is very high among addicts [28].

## 5. Conclusion

Patients with alcohol dependence are lacking in several components of EI. It might be effective to identify and train the individuals with lower scores in these aspects of EI to prevent alcohol related problems.

## Figures and Tables

**Table 1:** Demographic variables in control and alcohol dependent groups.

Variables	Control group		Alcohol dependent group	
	Number	Percentage	Number	Percentage
Education				
Diploma	28	70%	35	87.5%
Associate	7	17.5%	2	5%
BA/BS	4	10%	2	5%
MA/MS	1	2.5%	1	2.5%
Marital status				
Single	10	25%	8	20%
Married	28	70%	27	67.5%
Separated	2	5%	0	0
Divorced	0	0	2	5%
Widowed	0	0	3	7.5%
Occupation status				
Employed	27	67.5%	22	55%
Unemployed	7	17.5%	8	20%
Housewife	6	15%	10	25%
Type of admission				
Outpatient	38	95%	35	87.5%
Hospitalization	2	5%	5	12.5%
Smoking				
Used to do/I quit	5	12.5%	21	52.5%
Used to do/I do now	2	5%	15	37.5%
Never	33	82.5%	4	10%

**Table 2:** Emotional intelligence components in control group and alcohol dependent group and their comparison.

	Control group		Alcohol dependent group		Results of *t*-test		Effect size
	Mean	Standard deviation	Mean	Standard deviation	*t*-value	*P*	
Problem-solving	16.98	3.08	14.90	4.12	2.55	0.01	0.58
Happiness	17.63	3.68	16.95	3.49	0.84	0.40	0.19
Autonomy	19.63	3.40	17.25	4.19	2.79	0.007	0.63
Stress tolerance	21.38	3.45	19.52	3.52	2.38	0.02	0.53
Self-actualization	17.70	3.26	16.73	3.25	1.37	0.17	0.30
Emotional self-awareness	19.05	3.20	17.02	2.60	2.94	0.004	0.70
Realism	19.55	3.19	18.95	3.67	0.78	0.43	0.17
Interpersonal relations	16.95	3.59	15.80	4.02	1.35	0.18	0.30
Optimism	17.98	3.17	15.70	3.78	2.91	0.005	0.66
Self-esteem	18	3.17	15.97	3.61	2.67	0.009	0.60
Impulse control	21.55	3.76	18.67	5.22	2.83	0.006	0.64
Flexibility	20.90	3.87	19.15	2.83	2.31	0.2	0.52
Social responsiveness	20.65	1.72	11.45	3.40	15.26	0.001	3.59
Empathy	21.65	2.75	13.82	4.56	9.30	0.001	2.14
Self-expression	21.05	2.96	20.05	3.22	1.45	0.15	0.32

Total score	289	28.47	252	28.90	5.66	0.001	1.29

**Table 3:** Pearson correlation coefficient between components of emotional intelligence and the age of alcohol consumption in alcohol dependent individuals (*n* = 40).

Scales	Pearson correlation	Level of significance
Problem-solving	−0.19	0.23
Happiness	−0.10	0.51
Autonomy	0.12	0.48
Stress tolerance	**−0.38**	**0.02**
Self-actualization	−0.05	0.76
Emotional self-awareness	0.4	0.82
Realism	−0.26	0.10
Interpersonal relations	−0.11	0.49
Optimism	0.006	0.97
Self-esteem	−0.27	0.09
Impulse control	−0.23	0.16
Flexibility	0.16	0.34
Social responsiveness	−0.06	0.71
Empathy	0.23	0.16
Self-expression	−0.07	0.66

**Table 4:** Components of emotional intelligence in males and females with alcohol dependence.

	Male		Female		*P*
	Mean	Standard deviation	Mean	Standard deviation	
Problem-solving	16.22	4.03	15.65	3.51	0.50
Happiness	17.5	2.90	17.08	4.18	0.60
Autonomy	18.87	4.38	18	3.52	0.33
Stress tolerance	20.60	3.57	20.30	3.63	0.71
Self-actualization	17.72	3.30	16.73	3.21	0.17
Emotional self-awareness	**19.05**	**3.02**	**17.03**	**3.14**	**0.004**
Realism	19.52	3.09	18.98	3.76	0.48
Interpersonal relations	16.93	3.97	15.53	3.66	0.20
Optimism	**17.93**	**3.35**	**15.75**	**3.66**	**0.007**
Self-esteem	17.40	3.05	16.58	3.93	0.30
Impulse control	19.68	5.65	20.55	3.64	0.41
Flexibility	**21.15**	**3.21**	**18.9**	**3.42**	**0.003**
Social responsiveness	16.56	4.73	15.53	5.91	0.38
Empathy	17.60	4.73	17.88	6.11	0.82
Self-expression	20.53	2.72	20.48	3.49	0.83

Total	277.53	33.03	265.8	34.77	0.13

## References

[1] Sadock B. J., Sadock V. A., Ruiz P. (2009). *Kaplan & Sadock's Comprehensive Textbook of Psychiatry*.

[2] Kun B., Demetrovics Z. (2010). Emotional intelligence and addictions: a systematic review. *Substance Use & misuse*.

[3] Martins A., Ramalho N., Morin E. (2010). A comprehensive meta-analysis of the relationship between emotional intelligence and health. *Personality and Individual Differences*.

[4] Bastian V. A., Burns N. R., Nettelbeck T. (2005). Emotional intelligence predicts life skills, but not as well as personality and cognitive abilities. *Personality and Individual Differences*.

[5] Sudraba V., Rancans E., Millere I. (2012). The emotional intelligence features of substance use disorders patients: pilot research results. *International Journal of Collaborative Research on Internal Medicine & Public Health*.

[6] de Sousa Uva M. C., de Timary P., Cortesi M., Mikolajczak M., de Blicquy P. R., Luminet O. (2010). Moderating effect of emotional intelligence on the role of negative affect in the motivation to drink in alcohol-dependent subjects undergoing protracted withdrawal. *Personality and Individual Differences*.

[7] Samoei R. (2003). *Bar On Emotional Intelligance*.

[8] Sharifi V., Assadi S. M., Mohammadi M. R. (2007). Structured clinical interview for DSM-IV (SCID): Persian translation and cultural adaptation. *Iranian Journal of Psychiatry*.

[9] Ciarrochi J. E. (2001). *Emotional Intelligence in Everyday Life*.

[10] Saklofske D. H., Austin E. J., Galloway J., Davidson K. (2007). Individual difference correlates of health-related behaviours: preliminary evidence for links between emotional intelligence and coping. *Personality and Individual Differences*.

[11] Goleman D. (1995). *Emotional Intelligence*.

[12] Janati Y., Musavi A., Azimi H. (2010). Investigating emotional intelligence and self esteem level among nursing and midwifery students of Mazandaran University of Medical Sciences. *Journal of Mazandaran University of Medical Sciences*.

[13] Riley H., Schutte N. S. (2003). Low emotional intelligence as a predictor of substance-use problems. *Journal of Drug Education*.

[14] Brackett M. A., Mayer J. D., Warner R. M. (2004). Emotional intelligence and its relation to everyday behaviour. *Personality and Individual Differences*.

[15] Arya Sadr Z., Akbarzadeh N., Yazdi M. (2010). Compare addicted and non-addicted men components of emotional intelligence and educational programs based on the components of emotional intelligence in the city of Khorramabad. *Psychological Studies, Faculty of Education and Psychology, University of Al-Zahra*.

[16] Trinidad D. R., Johnson C. A. (2002). The association between emotional intelligence and early adolescent tobacco and alcohol use. *Personality and Individual Differences*.

[17] Trinidad D. R., Unger J. B., Chou C.-P., Johnson C. A. (2004). The protective association of emotional intelligence with psychosocial smoking risk factors for adolescents. *Personality and Individual Differences*.

[18] Trinidad D. R., Unger J. B., Chou C.-P., Azen S. P., Johnson C. A. (2004). Emotional intelligence and smoking risk factors in adolescents: interactions on smoking intentions. *Journal of Adolescent Health*.

[19] Parker J. D. (2006). Relationships with internet misuse, gaming abuse and emotional intelligence. *Personality and Individual Differences*.

[20] Taylor J. L., Kinderman P. (2002). An analogue study of attributional complexity, theory of mind deficits and paranoia. *British Journal of Psychology*.

[21] Zardkhaneh S. A., Rostami R., Zarean M. (2008). Emotional intelligence and defense mechanisms of addiction. *Iranian Journal of Psychiatry*.

[22] Petrides K. V., Frederickson N., Furnham A. (2004). The role of trait emotional intelligence in academic performance and deviant behavior at school. *Personality and Individual Differences*.

[23] Austin E. J., Saklofske D. H., Egan V. (2005). Personality, well-being and health correlates of trait emotional intelligence. *Personality and Individual Differences*.

[24] Bermas H. (2004). *Comparison of Self-Esteem, Attributional Style in Two Groups of Teenagers Addicted and Non-Addicted*.

[25] La Salvia T. A. (1993). Enhancing addiction treatment through psychoeducational groups. *Journal of Substance Abuse Treatment*.

[26] Dunn S. (2004). Emotional intelligence & addiction; 10 key points by Sussan Dunn, The EQ coach. *Journal of Substance Abuse Treatment*.

[27] Benjamin L., Wulfert E. (2005). Dispositional correlates of addictive behaviors in college women: binge eating and heavy drinking. *Eating Behaviors*.

[28] Clark L., Robbins T. W., Ersche K. D., Sahakian B. J. (2006). Reflection impulsivity in current and former substance users. *Biological Psychiatry*.

[29] Mayer J. D., Salovey P., Salovey P., Sluyter D. (1997). What is emotional intelligence?. *Emotional Development, Emotional Literacy, and Emotional Intelligence*.

